# A Lightweight Passive Human Tracking Method Using Wi-Fi

**DOI:** 10.3390/s22020541

**Published:** 2022-01-11

**Authors:** Jian Fang, Lei Wang, Zhenquan Qin, Bingxian Lu, Wenbo Zhao, Yixuan Hou, Jenhui Chen

**Affiliations:** 1 School of Software Technology, Dalian University of Technology, Dalian 116620, China; fangjian@mail.dlut.edu.cn (J.F.); qzq@dlut.edu.cn (Z.Q.); bingxian.lu@dlut.edu.cn (B.L.); houyixuan123@gmail.com (Y.H.); 2 The Key Laboratory of Ubiquitous Network and Service Software of Liaoning Province, Dalian 116024, China; 3 The Center of Underwater Robot, Peng Cheng Laboratory, Shenzhen 518066, China; 4 George R Brown School of Engineering, Rice University, Houston, TX 77005, USA; wz47@rice.edu; 5 Department of Computer Science and Information Engineering, The Artificial Intelligence Research Center, Chang Gung University, Kweishan, Taoyuan 33302, Taiwan, R.O.C.; 6 Center for Artificial Intelligence in Medicine, Chang Gung Memorial Hospital, Kweishan, Taoyuan 33375, Taiwan, R.O.C.; 7 Department of Electronic Engineering, Ming Chi University of Technology, Taishan District, New Taipei City 24301, Taiwan, R.O.C.

**Keywords:** channel state, device-free, RSSI, sensing, target tracking, Wi-Fi

## Abstract

Target tracking is a critical technique for localization in an indoor environment. Current target-tracking methods suffer from high overhead, high latency, and blind spots issues due to a large amount of data needing to be collected or trained. On the other hand, a lightweight tracking method is preferred in many cases instead of just pursuing accuracy. For this reason, in this paper, we propose a Wi-Fi-enabled Infrared-like Device-free (WIDE) method for target tracking to realize a lightweight target-tracking method. We first analyze the impact of target movement on the physical layer of the wireless link and establish a near real-time model between the Channel State Information (CSI) and human motion. Secondly, we make full use of the network structure formed by a large number of wireless devices already deployed in reality to achieve the goal. We validate the WIDE method in different environments. Extensive evaluation results show that the WIDE method is lightweight and can track targets rapidly as well as achieve satisfactory tracking results.

## 1. Introduction

Tracking moving objects to obtain their real-time location information and moving direction is a challenging issue [1]. Using wireless technology to achieve target tracking (i.e., a specific moving object for tracking) is an attractive method [2]. Using this method to perform accurate object tracking indoors is more challenging than tracking objects outdoors due to the complexity of the indoor environment, the multiple reflections at surfaces causing multipath propagation serving for uncontrollable errors, inevitable signal strength fluctuations, and so on [3]. Although many research works were investigated to overcome these issues, the drawbacks of these investigations are that they are too heavy and complex and thus are not suitable for real-time applications [4,5,6].

The development of communication technologies and the construction of cities (e.g., airports, shopping malls, and cell residential buildings with Wi-Fi infrastructure) provides an opportunity to multiplex Wi-Fi signals to scenarios without deploying additional hardware. They solve the problem of blind spots and greatly reduce overhead, and they have achieved high accuracy due to the ubiquitous and abundant features of Wi-Fi signals [7]. However, wireless indoor positioning methods usually need to use the receiver’s wireless signal reception status for positioning. This requirement inevitably limits the practical use of wireless indoor positioning.

An attractive technology, Device-free Localization (DfL), without asking for any accessories on the targets, has attracted the attention of researchers due to its applicability and flexibility [8]. Traditional Received Signal Strength (RSS)-based DfL methods [9,10] are usually coarse-grained and limited by the multipath effect, incurring unsatisfactory localization accuracy. Other works either build a complex Channel State Information (CSI) fingerprint database [11] or a map from the location of the target to CSI dynamics [12]. Although they increase the accuracy, they sacrifice resources due to their high overhead and repeated process for collecting data in dynamic scenarios. In addition, offline training requires a lot of time, which makes it fail in real-time practices. Some AI methods make it possible to be less sensitive to the environment and people when collecting the data, so that training problems can be solved in the lab in one go, but they are still mostly limited to the field of action recognition, and there is still research to be done for continuous large-range tracking.

In some large sensory environments (e.g., museums or airports), the existing Wi-Fi facilities creates a large number of links, each with a different background environment. Here, we propose a Wi-Fi-enabled Infrared-like Device-free (WIDE) target-tracking method by leveraging these existing Wi-Fi links. We first propose a concept of using the line-of-sight (LoS) path between a pair of Wi-Fi transceivers to form an enhanced and featured infrared-like beam with a Fresnel zone. A lot of research shows that differences in the characteristics of wireless signals are obvious when there is a block on the LoS path of the link [13]. Therefore, we set an universal threshold so that links with different background environments can accurately determine the block of LoS by target movement. Then, with enough links, we can form a Wi-Fi-enabled infrared-like grid environment to achieve a device-free indoor target tracking by identifying targets when and how to go through the LoS.

To enable the WIDE method, we need to solve several issues. First, the WIDE method cannot rely too much on scenario-customized calibration. Secondly, it faces challenges such as the setting of thresholds in different environments, even though none of the human activities are present. Thirdly, since the CSI sequences can exhibit different amplitudes and different background noises can lead to different signal fluctuations, the WIDE method needs to achieve a uniform threshold. Fourthly, it needs to consider how to distinguish whether the signal changes due to human activities or abnormal signals due to objects falling and the different effects of people moving at different speeds. Fifthly, it also needs to know how to distinguish a stationary target within the sensing range without relying on the training method when the effect of stationary targets on the signal is relatively weak.

The WIDE method does not request any complex fingerprint map information for indoor localization. We utilize *phase difference* as the metric to track the route of one or more targets in near real time. The main contributions of this paper are as follows.

1. We are the first to propose using the LoS path between a pair of Wi-Fi transceivers to form an enhanced and featured infrared-like beam with a Fresnel zone.
2. A data stream and subcarrier selection algorithm is proposed to reduce the loss of effective characteristics while maintaining the computational effort for supporting near real-time tracking.
3. A comprehensive study of environment-adaptive thresholds of eigenvalues of the environment is presented for the recognition of a target crossing the link in different environments. Based on this, we are the first to propose a tracking method based on a Wi-Fi grid that achieves a near real-time, meter-level tracking under the condition of a limited number of transceivers.

The rest of this paper is organized as follows. In Section 2, we review the related research progress in a categorized manner. Section 3 describes how we acquired and analyzed the data, built the model, calculated the thresholds, and evaluated the accuracy. Section 4 describes how we make full use of the existing equipment to build the grid and implement the target tracking, and it also gives an analysis of the error and robustness. Finally, we conclude our works and discuss possible future works in Section 5.

## 2. Related Works

### 2.1. Human Activities Sensing

Typical application scenarios in the work of human sensing using Wi-Fi signals are the detection of some daily behaviors, such as standing, sitting, lying down, walking, running, and so on. Some works define some combined behaviors, such as identification by detecting a series of habitual behaviors when the target returns home, such as changing shoes first and then putting on clothes. With the development of signal processing and artificial intelligence technology, some more tiny movements can also be recognized and utilized.

Early human activity sensing works were mostly based on camera video [14] and infrared [15]. In recent years, there has been an increase in wireless sensing using Wi-Fi. The concept of passive human detection was first proposed in [16], which used the moving average and moving variance of Received Signal Strength Indication (RSSI) values based on sliding windows to determine a threshold value and the presence of a target.

Pu et al. identified several actions to achieve new human–computer interaction by extracting action-related Doppler shift features from Wi-Fi signals [17]. In recent years, with falls becoming the biggest threat to the health of the elderly, more and more work is focusing on home monitoring; the article [18] achieved 90% high-accuracy single-person fall detection using the temporal stability and frequency diversity of CSI. The article [19] improved the sensitivity by 14% and found that the phase difference is a more sensitive feature for activity recognition, enabling real-time activity segmentation for the classification of falls and fall-like behaviors, solving the inherent drawback of previous work that makes practical deployment difficult due to the assumption of natural segmentation between activities.

Qian et al. obtained the original phase by linearly transforming the original data to remove noise and found it to be more sensitive to human activity, and the method was robust even when the target is moving [20]. Zhou et al. considered the impact of Wi-Fi coverage on sensing and proposed the Omnidirectional Passive Human Detection (Omni-PHD) method [21] using the multipath effect to achieve the omnidirectional human detection. Another system that leverages changes in WiFi signal strength to sense in-air hand gestures around the user’s mobile device called WiGest [22] achieves gesture recognition by extracting the rising edge and falling edge from RSSI of Wi-Fi without training. Its results showed that it achieves good accuracy in wall-through non-visual scenarios. Sun et al. [23] introduced a signal angle-of-arrival model to track the movement of target fingers to achieve recognition of users writing in the air. Wang et al. [24] achieved lip recognition, making a breakthrough in the field of tiny activity recognition and proving the potential of CSI for wireless sensing.

The wireless sensing technology is not only used in tracking locations but also used for medical healthcare. Wang et al. implemented the monitoring of both respiratory rate and heartbeat using CSI-corrected phase difference information to achieve a comprehensive assessment of the target sleep quality [25]. The work [26] used the periodic level of CSI sequences as a feature to detect sleep quality and could well distinguish whether the change in CSI data was caused by sleep posture change or sleep apnea to achieve abnormal breathing tracking. Other works [27,28,29] achieve single to multi-person respiration monitoring using a Fresnel model, etc., allowing wireless sensing to move to a more subtle level. In recent years, AI techniques [30,31,32,33,34,35] have been introduced into the field of wireless sensing to achieve improved accuracy by building novel datasets and constantly updating various training methods. However, these methods generally have the problem of not being interpretable and require a certain amount of overhead and time for both collecting and training data, which does not fit the context of this study.

### 2.2. Device-Free Tracking

The energy-efficient framework for high-precision multi-target adaptive DfL approach (E-HIPA) [36] and the fine-grained and low cost DfL approach (FitLoc) [9] applied compressive sensing to localize one or more targets with very little RSS data and human efforts. A real-time, accurate, and scalable system (Rass) [37] established the relationship between signal fluctuations and the divided triangular areas.

The development of wireless physical layer research has led to more researchers focusing on CSI-based positioning methods. Wu et al. proposed a real-time LoS identification scheme called PhaseU [13] in various scenarios that requires the user to bring sensors. A Wi-Fi-based decimeter-level tracking system (Widar) [12] estimates velocity and location by modeling CSI dynamics without statistical learning. The model-based DfL system (LiFS) [3] finds the subcarrier least affected by multipath effects and calculates a set of power fading equations to determine the target’s location. The device-free indoor human tracking system (IndoTrack) [2] proposes Doppler-MUSIC and Doppler-AoA methods to extract and estimate velocity and location information from CSI with only commodity Wi-Fi. Zhang et al.  [38,39,40] first introduces the Fresnel zone concept into passive human sensing and obtains fine-grained respiration detection and localization results. Their work explained the phenomenon that the performances of different positions are significantly different theoretically. The articles [14,41] tried to integrate as many as possible technologies to obtain a better tracking effect. Recently, some works explored the possibility of using LPWAN for a larger range of sensing and positioning [42,43,44].

## 3. Data Acquisition, Processing, and Model Building

Based on previous work [45], we first propose an algorithm based on CSI sliding variance to determine whether there are moving targets within the sensing range and count the periods when human activities exist. To improve the accuracy, we consider the amplitude and the phase difference and then design a set of filters, including outlier removal, linear interpolation, wavelet denoising, etc. We also design methods to select data streams and subcarriers to adapt to the occasional instability of the CSI.

### 3.1. Feature Extraction and Performance Analysis

**Figure 1:**
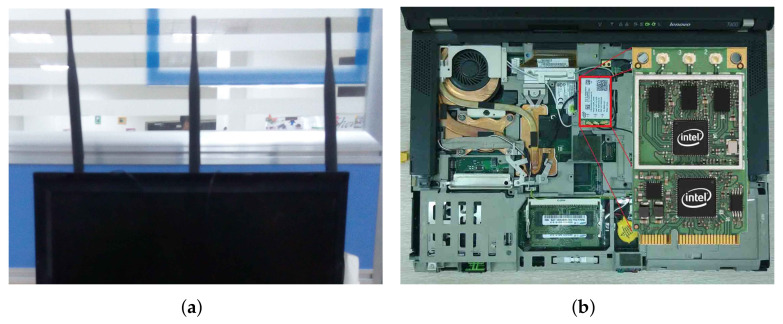
Devices used in the experiments. (**a**) Three antenna receiver. (**b**) Intel 5300 NIC.

The initial acquisition of CSI is completed by calling the functions provided by the Linux 802.11n CSI Tool [46]. The transmitting frequency is set to 30 Hz. The complex matrix CSI (1 × 3 × 30, 1 is the number of transmitting antennas, 3 is the number of receiving antennas, and 30 is the number of subcarriers) carries a large amount of information reflecting the characteristics of the environment. Since one transmitting antenna and three receiving antennas are used, the CSI matrix can be split and reorganized into three data streams, each containing 30 subcarriers, which are used to observe the time domain characteristics of each of the three links. The devices used in the experiments are shown in Figure 1.

#### 3.1.1. Extraction of Amplitude and Phase Difference

**Figure 2:**
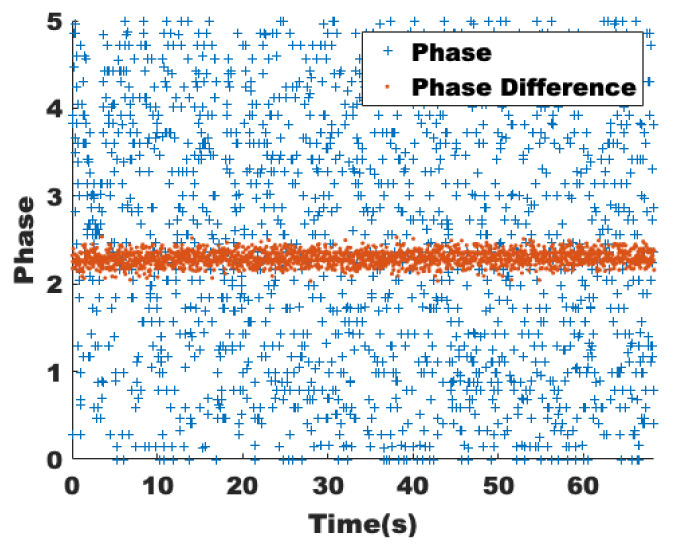
Raw phase and phase difference without human presence.

Considering the background meaning and the data-length requirement of wavelet transform and Fourier transform, the middle 2048 packets are selected for each data stream, which corresponds to a time of about 68s. The amplitude and phase can be obtained from the complex matrix by transformation. It is first observed experimentally how the amplitudes vary for different data streams and different subcarriers as in [18], where human activity independently affects different data streams, while the effects on different subcarriers of the same data stream are similar. Although CSI is widely used in the field of environmental sensing, most of the related work has only used amplitude, and the phase is greatly limited due to the clock synchronization errors that cause phase shifts. The MIMO technology can eliminate the problem by using multiple antennas. As shown in Figure 2, the original phase and phase difference of the tenth subcarrier of a data stream are extracted from the CSI collected in a static environment. The effect on the phase and amplitude of different data streams and different subcarriers by human activity is the same, so the selection can be done in such a way as to retain comprehensive information while significantly reducing the number of operations.

Since the multi-antenna receiver uses the same sampling clock, the difference in the relative error between every two antennas is fixed despite the random sampling error generated at different moments for each antenna. Thus, the measured phase difference $\Delta \hat{\phi_{k}}$ can be calculated as

(1) $$\begin{array}{cc} \Delta \hat{\phi_{k}} & =\hat{\phi_{1,k}}-\hat{\phi_{2,k}}\\ \; & =\left(\phi_{1,k}-\phi_{2,k}\right)+2\pi \frac{k}{N}\left(n_{1,\varepsilon }-n_{2,\varepsilon }\right)+\left(\beta_{1}-\beta_{2}\right)\\ \; & =\Delta \phi_{k}+2\pi \frac{k}{N}\delta n_{\varepsilon ,CSI}+\Delta \beta , \end{array}$$

where $\Delta \phi_{k}$ is the real phase difference, i=1,2 represent the two antennas used to calculate the phase difference, *k* represents the number of the subcarrier, $n_{i}\varepsilon$ represents the clock synchronization error of each of the two antennas, and $\beta_{i}$ represents the constant error. Although Δβ takes different values at different times, we can use the cyclic nature to shift the phase so that it takes the same value at different times. We can assume that its value is 0. Then, Equation (1) can be rewritten as

(2) $$\Delta \hat{\phi_{k}}=\Delta \phi_{k}+2\pi \frac{k}{N}\Delta n_{\varepsilon ,CSI}.$$

When the channel state is stable, the $\Delta n_{\varepsilon ,CSI}$ also remains unchanged, $\Delta n_{\varepsilon ,CSI}=\frac{d\sin\theta }{cT_{s}}$, where θ is the angle of incidence of the signal, $T_{s}$ is the sampling interval, and λ is the wavelength. According to the channel independence property of MIMO technology, the minimum value of *d* is $\frac{\lambda }{2}$; then, we have $\Delta n_{\varepsilon ,CSI}\leq \frac{1}{2fT_{s}}$, and *f* is the center frequency of the carrier (2.4 GHz), and $T_{s}$ is 50 ns as an experience value. We get $\Delta_{\varepsilon ,CSI}\leq 0.0083$, $-0.0262\leq 2\pi \frac{k}{N}\Delta_{\varepsilon ,CSI}\leq 0.0254$, which can be neglected, that is $\Delta \hat{\phi_{k}}≃\Delta \phi_{k}.$

The method of feature extraction is described in Algorithm 1.
**Algorithm 1:** Feature extraction.**Input:**     data_file**Output:**     amp, phase_diff  1: original_trace←***read_bf_file***(data_file);  2: sqeezed_trace←***get_sqeezed***(original_trace);  3: csi_trace←***change_length***(sqeezed_trace);  4: Get timestamp and calculate interpolation length;  5: **for** i← to ***size***(csi_trace) **do**  6:     csi_entry←csi_trace(i);  7:     csi(i)←***get_scaled_csi***(csi_entry);  8: **end for**  9: abs_amp←abs(csi);10: amplitude←***interp***(csi, len);11: amp←***center_data***(amplitude);12: rx1_ph←***angle***(rx1_csi);13: rx2_ph←***angle***(rx2_csi);14: diff←***unwrap***(rx1_ph) - ***unwrap***(rx2_ph);15: ph_diff←***warptopi***(diff);16: phase_diff←***interp***(ph_diff, len);

It is concluded that the phase difference eliminates the phase shift caused by the time synchronization error and is only related to the channel state; thus, the amplitude and phase difference are chosen as the features. The stability of amplitude and phase to the static environment, sensitivity to human activities, and robustness to line-of-sight and non-line-of-sight paths will be verified by the following experiments.

#### 3.1.2. Sensitivity and Robustness Analysis

**Figure 3:**
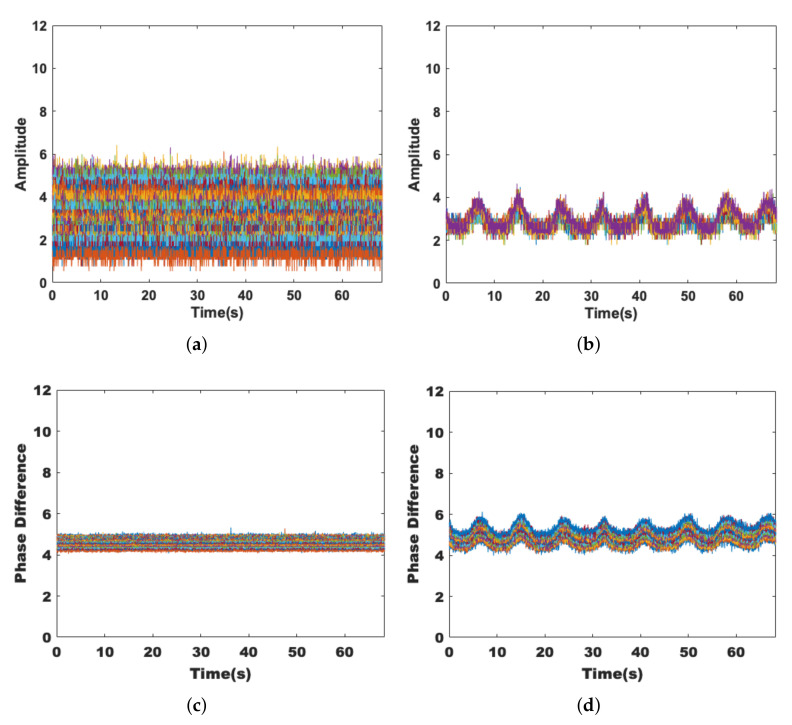
Sensitivity of amplitude and phase difference. (**a**) The amplitude of the static environment. (**b**) The amplitude with target breathing. (**c**) The phase difference of the static environment. (**d**) The phase difference with target breathing.

To achieve a better detection result, we hope that the extracted features can show good stability in a static environment while showing sufficient sensitivity to the presence of human activities. Figure 3a,c are data collected in an empty room, while Figure 3b,d are data collected with the volunteer sitting near the receiver; it can be seen that left figures tend to be smooth, while the right figures show regular changes. This is because the chest cavity moves back and forth regularly when breathing, and the channel state also changes, which is manifested in the amplitude and phase difference as obvious wave peaks and troughs, and it verifies the sensitivity of these two features to tiny human activities.

**Figure 4:**
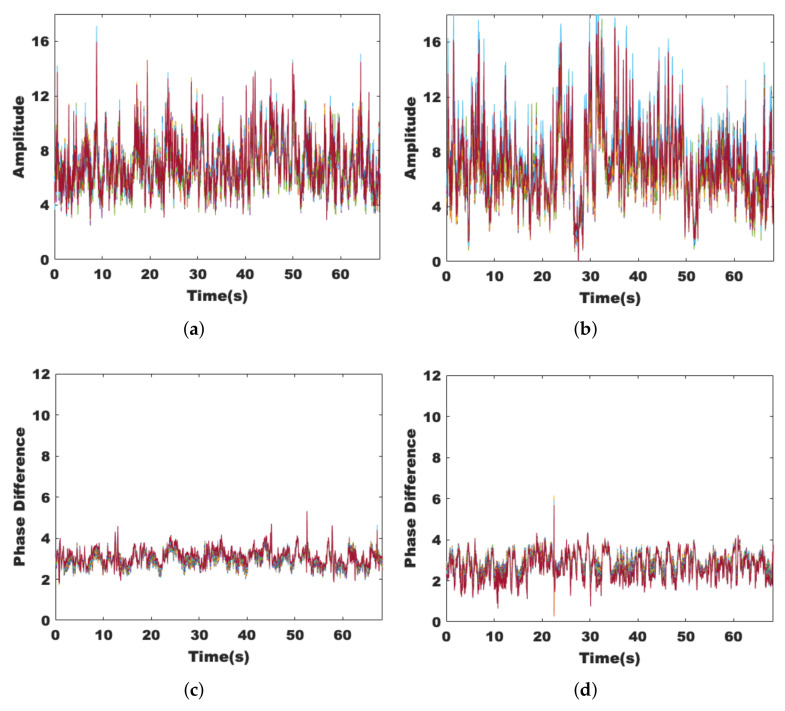
Robustness of amplitude and phase difference. (**a**) The amplitude under nLoS. (**b**) The amplitude under LoS. (**c**) The phase difference under nLoS. (**d**) The phase difference under LoS.

Since human activities may occur everywhere, we conduct experiments under both nLoS and LoS paths and collect data for 60 s each. Figure 4a,c show the effects on the amplitude and phase difference when the volunteer is active on the nLoS path, respectively, while Figure 4b,d show the effects on the signal when the volunteer is active on the LoS path. It can be seen that the signal fluctuations are more pronounced when human activity occurs on the LoS path. However, they are still robust on the nLoS path.

#### 3.1.3. Dimensionality Reduction of Data Streams and Subcarriers

**Figure 5:**
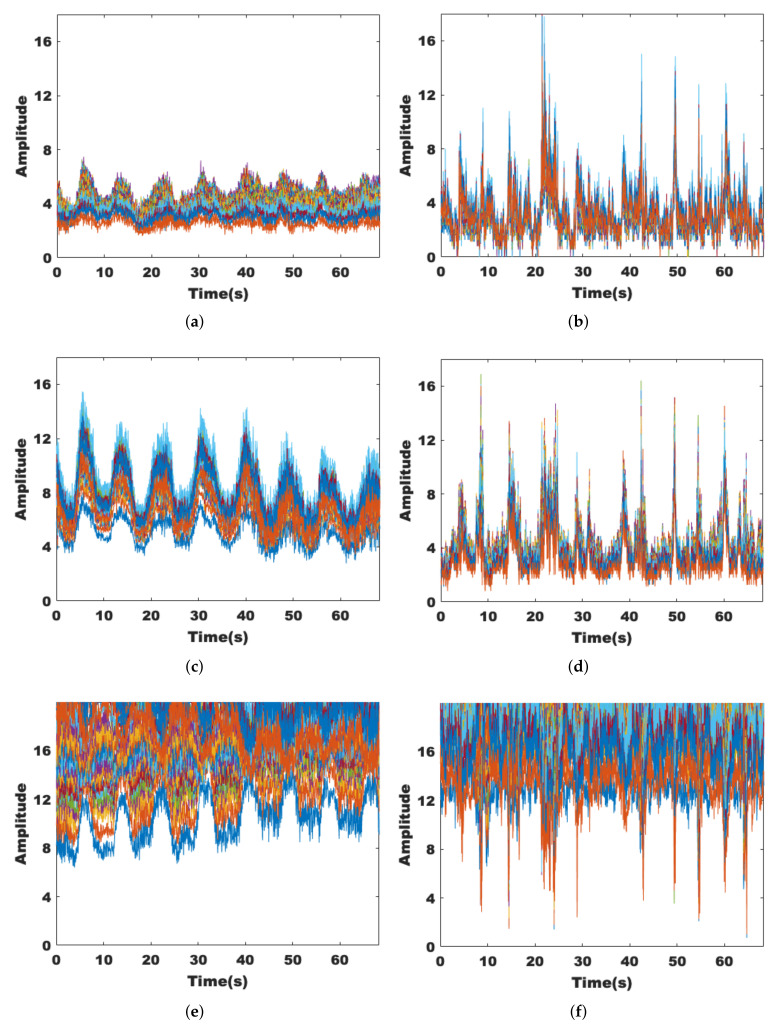
Performance of different data streams. (**a**) With target breathing, Rx = 1. (**b**) With target moving, Rx = 1. (**c**) With target breathing, Rx = 2. (**d**) With target moving, Rx = 2. (**e**) With target breathing, Rx = 3. (**f**) With target moving, Rx = 3.

Although the MIMO technique allows for more fine-grained environmental sensing, the increase in the amount of CSI data leads to a significant increase in computation. Therefore, the CSI data should be downscaled first. We find that in most cases, the data streams with intermediate amplitude size describe the channel state more accurately. Figure 5a–f indicate the different trends of the three data streams when someone is breathing and walking within the sensing range, respectively. The data stream with the middle amplitude value shows relatively stable fluctuations and is also more sensitive to the presence of human activities.

The center frequencies of 30 subcarriers are different; there will be frequency-selective fading in the face of multipath effects. If features are extracted from only one subcarrier, it will lead to inaccurate environmental sensing. If the features are extracted for all 30 subcarriers, it will make the computation too large. These subcarriers exhibit some correlation with each other; therefore, the adjacent subcarriers can be downscaled by principal component analysis (PCA). The main idea of PCA is to map the n-dimensional features to the orthogonal k-dimensions by finding a set of mutually orthogonal axes in the original space, and the k axes contain most of the variance and the rest contain almost zero variance. The reconstructed *k*-dimensional features are called principal components.

**Figure 6:**
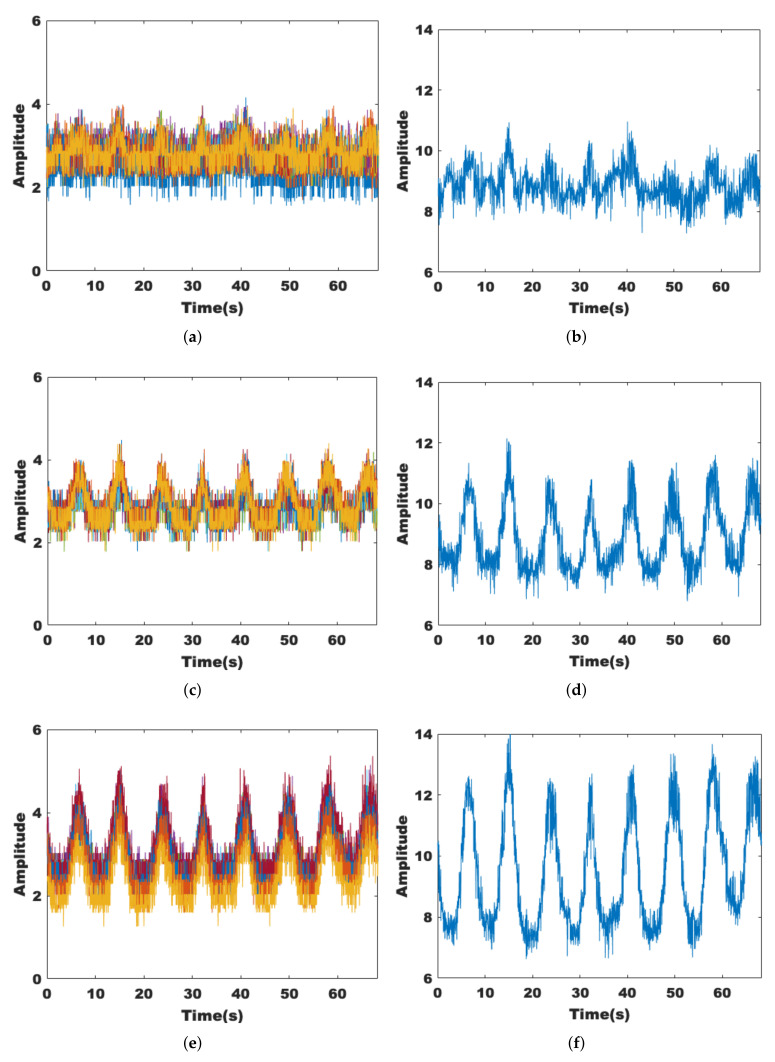
Performance of different data streams. (**a**) Rx = 1, subcarrier 1:10. (**b**) Principal component analysis for subcarriers 1 to 10. (**c**) Rx = 1, subcarrier 11:20. (**d**) Principal component analysis for subcarriers 11 to 20. (**e**) Rx = 1, subcarrier 21:30. (**f**) Principal component analysis for subcarriers 21 to 30.

Figure 6a,c,e show 30 subcarriers of the same data stream in groups of 10. It can be seen that neighboring subcarriers in the same group show similar transformation trends, while different groups are different. Figure 6b,d,f show the results of dimensionality reduction for each of the 10 subcarriers, and it can be seen that the dimensionality of the processed data has been reduced while retaining the original features.

### 3.2. Data Pre-Processing

#### 3.2.1. Removing Outliers

**Figure 7:**
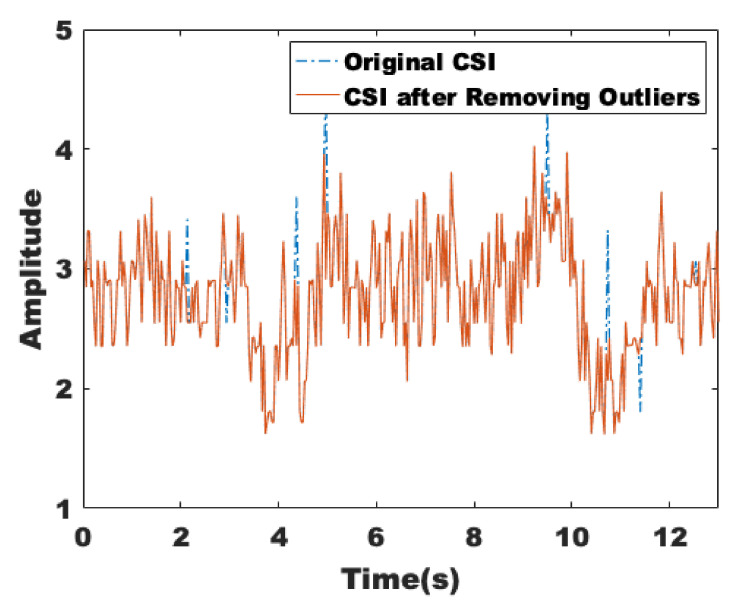
The CSI after removing outliers.

Then, we need to pre-process the CSI data and eliminate the outliers. The Hampel filter has two parameters that specify the number of samples k and several times the standard deviation (N) sigma on both sides of each sample in the window. If the difference between the value of the sample and the median is more than nsigma times of the standard deviation, the sample is replaced with the median. In this paper, Hampel filtering is applied to all subcarriers, and Hampel treats each column of the CSI matrix as a separate channel. As shown in Figure 7, there are some obvious abrupt change points around 4.3 s and 8.9 s, and the red curve is the result after removing the identified outliers.

#### 3.2.2. Linear Interpolation

Although the sending device is set to 30 packets/s, there is no guarantee that packet loss will not occur. Since multiple devices share the Wi-Fi channel, it may lead to an uneven interval of received packets. To make the horizontal axis corresponding to the timestamp also equally spaced and make the samples evenly distributed, the number of packet loss is first calculated by the timestamp, and then, one-dimensional linear interpolation is performed on the CSI.

#### 3.2.3. Wavelet Denoising

For a normal human event-related signal, knowing only which frequency components it contains is not enough to determine the beginning and end of the event that caused the signal to change; it is also necessary to know how the frequency of the signal changes over time, which is also called time-frequency analysis.

In contrast with Short-Time Fourier Transform (STFT), the wavelet transform not only retains the localization but also can change the shape of the window and spectral structure by adjusting the size of the scale parameter, playing a “zoom” role. As can be seen from Equation (3), the left Fourier transform has only one variable frequency ω, while the wavelet transform on the right of the arrow has two: scale α and translational volume τ, where α is used to control the scaling of the wavelet function, and τ determines the translation of the wavelet function.

(3) $$\begin{array}{c} \begin{array}{cc} F(\omega ) & =\int_{-\infty }^{+\infty }f\left(t\right)\times e^{-i\omega t}dt\\ \Rightarrow WT(\alpha ,\tau ) & =\frac{1}{\sqrt{\alpha }}\int_{-\infty }^{+\infty }f\left(t\right)\times \psi \left(\frac{t-\tau }{\alpha }\right)dt \end{array} \end{array}$$

**Figure 8:**
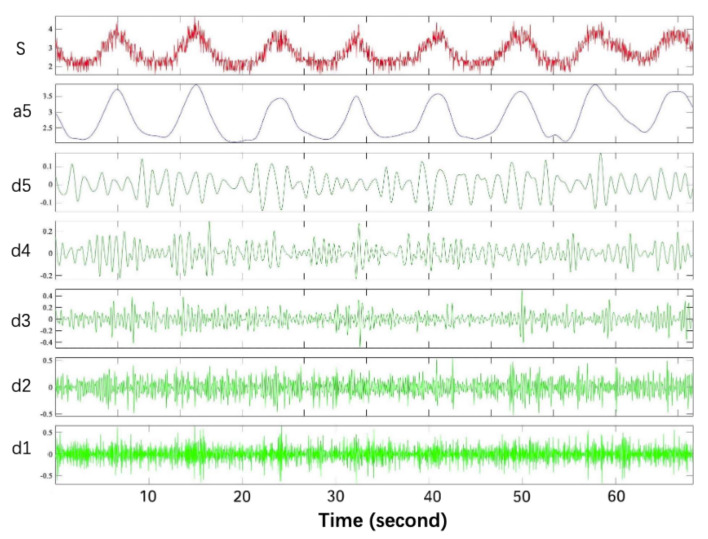
Wavelet domain denoising.

We use the DWT filtering, as shown in Figure 8, where s is the noise signal, d1, d2, d3, d4, and d5 are the high-frequency coefficients, and a5 is the low-frequency coefficient.

Since CSI is noisy in all frequency bands, an in-band noise filtering technique, discrete wavelet transform, is chosen in this paper. Through the careful selection of parameters, the in-band noise is eliminated while retaining the high-frequency components to reduce signal distortion. By using the characteristics of wavelet transform translation and scaling, the signal is filtered by constructing a finite-length wavelet basis that will decay, which can not only obtain the frequency of the signal but also locate the time when the frequency components appear and remove the noise by multi-resolution analysis. A noise-containing model is represented as

(4) S(k)=f(k)+ε×e(k),k=1,2,⋯,n−1,

where S(k) is the signal affected by the noise, f(k) is the useful signal, e(k) is the noise, and ε is the standard deviation of the noise coefficient. Normally, the f(k) behaves as a smooth signal at low frequencies, while the noise e(k) fluctuates more and has a higher frequency. The purpose of wavelet noise reduction is to remove the noise e(k) and recover the useful signal f(k). In general, the noise reduction of a one-dimensional signal is divided into three steps.

**Figure 9:**
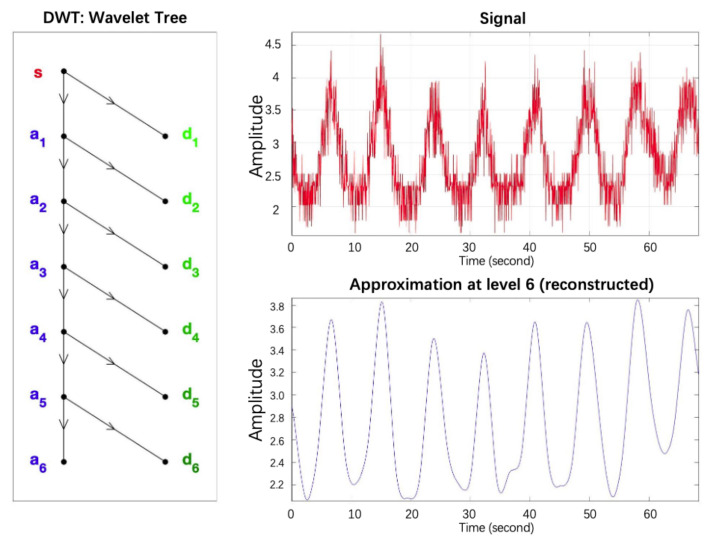
Wavelet decomposition tree.

(1) Wavelet decomposition of the signal. Firstly, we need to select a wavelet basis function.
  The sym wavelet is an improvement of the db wavelet, which has better symmetry while retaining better regularity, so we choose sym8 as the wavelet basis function. Next, the number of layers N to be decomposed is determined. Considering the results of experimental observation, N is set to 6; then, we do the 6-layer wavelet decomposition to S(k) according to the wavelet decomposition tree shown in Figure 9.
(2) Threshold quantization of high-frequency coefficients. Determine a suitable threshold value to quantize the high-frequency coefficients of each layer. The main processing methods are hard-threshold and soft-threshold quantization. In this paper, the soft threshold function is chosen because the processing of the signal is relatively smooth. The soft threshold function means that when the absolute value of the wavelet coefficients is less than the given threshold, let it be 0. When it is greater than the threshold, let it be minus the threshold.
  (5) $$w_{\lambda }=\left\{\begin{array}{c} {}[sign(w)](|w|-\lambda ),|w|\geq \lambda \\ 0,|w|<\lambda \end{array}\right.$$
  where *w* is the wavelet coefficients, wλ is the wavelet coefficients after applying the threshold, and λ is the threshold value.
(3) Wavelet reconstruction of the signal. Based on the high-frequency coefficients of the N layer and the low-frequency coefficients of the N${}_{th}$ layer after the second quantization step, wavelet reconstruction is completed to remove the noise and recover the useful signal.

### 3.3. Environmental Adaptive Mechanism Based on Eigenvalue Density Estimation

Passive human detection belongs to the detection of anomalies for signal processing, because usually, the signal acquired in a static environment tends to be stable, while the signal exhibits significant fluctuations when there is human activity. Therefore, the background data in the static environment need to be collected first, and other modules will rely on it to detect anomalies. If the observed value corresponds to a feature value exceeding the threshold, it is considered that human activity may have caused the fluctuation of the signal. When the transceiver device is deployed in different scenarios or the background environment changes with time, the threshold value will also change. Therefore, we propose an environmental adaptive mechanism to adjust the features extracted from the observations in real time by referring to the data collected in a static environment, so that they can remain stable when the environment changes. The mechanism is two-fold. Firstly, when the system is first started, it is required that no target exists, and the module needs a short phase to complete the initialization, generate the configuration file corresponding to the static environment, and save the feature values corresponding to the static environment. Subsequently, due to the dynamic changes of the environment, the feature values may not represent a real state of the environment at this time. Therefore, the module keeps the static environment configuration file updated in real time to adapt to the changes in the environment. The definition of the sliding window used in the following is given: there are 30 subcarriers, *k* is the number of the subcarrier, and *l* is the length of the sliding window. $S_{k,t}$ represents the amplitude of the *k*th subcarrier at time *t*. The corresponding sliding window can be expressed as $W_{k,t}=\left[S_{k,t-l+1},S_{k,t-l+2},\cdots ,S_{k,t}\right]$. Then, the features $x_{k,t}$ are extracted from each sliding window in turn. When the feature to be extracted is the mean value, we have

(6) $$x_{x,t}=g\left(W_{k,t}\right)=\frac{1}{l}\sum_{l}^{i=1}S_{k,t-l+i}.$$

At initialization, the module extracts the eigenvalues from the collected data sequentially based on a sliding window and uses the estimated probability density function (PDF) to determine the eigenvalues corresponding to the static environment. We use kernel density estimation (KDE), which is a nonparametric method for estimating PDF. When the $k_{th}$ subcarrier is estimated, a set of sliding windows is first obtained, the number of which is *n*, and the length of each window is *l*. Next, for each window, we use the function $g(W_{k,i})$ to extract the eigenvalues and obtain the eigenvalues $x_{k,i}$ corresponding to the window $W_{k,i}$. Assume that $f_{k}$ is the set of observations just calculated by $x_{k,i}$ where *i* is the PDF from 1 to n, $f_{k}$ can be obtained from the KDE method by expressing it as

(7) $$\hat{f_{k}\left(x\right)}=\frac{1}{nh_{k}}\sum_{i=1}^{n}V\left(\frac{x-x_{k,i}}{h_{k}}\right),$$

where *V* is the kernel function Epanechnikov, which is optimal in the sense of mean square error. $h_{k}$ is the smoothing parameter, which is often called the bandwidth or window. We refer to the work [47] to estimate the optimal bandwidth

(8) $$h_{k}=2.345\hat{\sigma_{k}}n^{-0.2},$$

where $\hat{\sigma_{k}}$ is an estimate of the standard deviation of the observed value $x_{k,i}$. After the estimation of the PDF, it is used to determine the eigenvalues of the static environment and is saved in the static profile, which is defined as $\hat{F_{k}^{-1}}\left(1-\alpha \right)$, where $\hat{F_{k}}$ is the cumulative distribution function (CDF) of $\hat{f_{k}}$. It also represents the upper bound of the standard deviation, and if the observed values exceed this value, they will be considered as outliers. After the initialization, the environment adaptive module also updates the static environment profile as the environment changes. After comparing the feature values of the sliding window with the static environment profile, if it is determined that no target exists in the sensing range for 10 s, the PDF is re-estimated by adding the eigenvalues of the sliding window. Since the environment change over time, the newer the data, the higher the weights that should be assigned when performing the estimation $w_{i}$, and we have $\sum_{i=1}^{n}w_{i}=1$, where the  weight of linear variation is $w_{i}=\frac{i}{n(n+1)/2}$. The equation used to estimate the PDF is given as

(9) $$\hat{f_{k}\left(x\right)}=\frac{1}{h_{k}}\sum_{i=1}nw_{i}V\left(\frac{x-x_{k,i}}{h_{k}}\right).$$

Then, we recalculate the value of $\hat{F_{k}^{-1}}\left(1-\alpha \right)$ and update the configuration file of the static environment. The algorithm of the environmental adaptive mechanism is as shown in Algorithm 2.
**Algorithm 2:** Environmental adaptive mechanism.**Input:**     amp_seq**Output:**     sta_profile  1: ***Initialization:*** the feature of current environment  2: Get value from the initialized sta_profile: $\sigma_{static}$;  3: flag← ***mod_select***(amp_seq);  4: **if** flag=true **then**  5:      **for** i← to length(amp_seq)/15 - 1 **do**  6:        amp_window← amp_seq (i×15+1: i×15+15);  7:        feature←$\sigma_{amp\_window}/\sigma_{static}$;  8:        **if** feature < threshold **then**  9:           add feature to fea_set;10:      **end if**11:    **end for**12:    $\hat{f_{k}\left(x\right)}$← ***Epanechnikov***(fea_set);13:    $\sigma_{static}$←$\hat{F_{k}^{-1}}\left(1-\alpha \right)$;14:    sta_profile←$\sigma_{static}$;15: **end if**

### 3.4. Module Selection

In this paper, data were collected for unoccupied scenes, in the presence of stationary targets, and with targets moving, including stationary targets standing, sitting, lying down, etc., as well as dynamic targets moving at different speeds across the LoS path, respectively. The presence of stationary targets and unoccupied scenes are categorized as cases without target movement. Since although CSI is also sensitive to chest motion, its fluctuations are significantly larger when there are moving targets. We use a sliding window-based approach and select robustness features and environment adaptive thresholds to distinguish the two cases, and we also design methods to distinguish the effect of real human activities and other noises such as falling objects. We use the variance of the signal to describe the fluctuation of the signal, which is mathematically represented as the standard deviation. However, as the environment changes, the threshold value will also change, and the location of the transceiver will affect the threshold value; i.e., the threshold value obtained by experimental calibration in one environment cannot be directly applied to other environments, limiting the deployment of multiple links. Therefore, in this paper, the ratio between the standard deviation of the sliding window $\mu_{now}$ and the standard deviation of the static environment $\sigma_{static}$ is used as a robustness feature as shown in Equation (10). As introduced above, when the system is first started, a short phase is required to initialize the static environment profile for each link and per stream and subsequently update it in real time. That is, when the environment changes, it is only necessary to adjust the static environment’s configuration file by the adaptive module:

(10) $$\mu_{now}=\frac{\sigma_{now}}{\sigma_{static}}.$$

After the collected CSI data are pre-processed, a threshold-based approach is first used to roughly determine if there are fluctuations. If not, the environment adaptive module will update the static environment profile to adapt to the changes in the environment. When there are fluctuations, it may be caused by falling objects or pets. However, the time they take is always much less than the time it takes for a person to cross the sensing range. If the signal fluctuation is caused by a falling object, the static target detection module is invoked to analyze it and further determine whether a stationary person is present. To avoid calling the wrong module due, we will double-check the data located in the critical area to prevent missing the static targets that may exist in the sensing range. Although the normalized standard deviation-based method can only give a speculative conclusion about the presence of moving targets, it is very lightweight and allows for fast and efficient computation, and a finer-grained detection method will be proposed as follows.

### 3.5. Anomaly Detection

**Figure 10:**
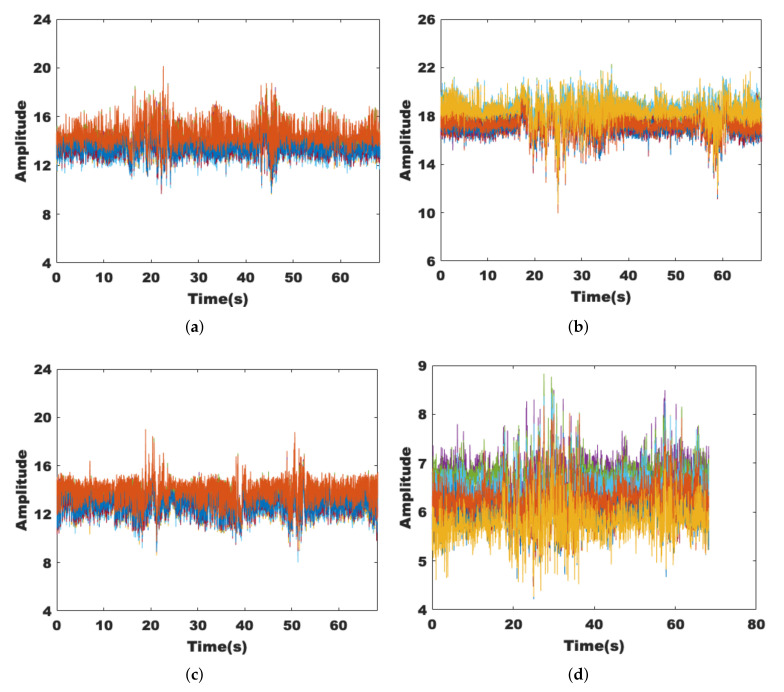
Sensing under LoS and nLoS condition: Fast (0.6 m/s) and Slow (0.3 m/s). (**a**) Fast passage of targets under nLoS. (**b**) Slow passage of targets under nLoS. (**c**) Fast passage of targets under nLoS. (**d**) Slow passage of targets under nLoS.

We design a basic anomaly detection module to process the data to detect anomalous changes in the signal caused by human activities, as shown in Figure 10 for the effects of the target passing through the sensing range at different speeds under LoS and nLoS conditions, respectively. The evaluation of the anomalies relies mainly on the eigenvalues saved in the profile about the static environment mentioned above. For the data related to the *k*th subcarrier, first calculate the sliding window $W_{k,t}$ corresponding to the eigenvalues $x_{k,t}$, and calculate the anomaly score for each sliding window $\alpha_{k,t}=\frac{x_{k,t}}{\mu_{k}}$, where $\mu_{k}=\hat{F_{k}^{-1}}\left(1-\alpha \right)$, which is the feature value of the static environment stored in the configuration file. The anomaly score will exceed 1 when the window has abnormal fluctuations, and because the general indoor environment is noisy and the signal inevitably has abnormal fluctuations, if the period is marked as abnormal based on a single data stream and single sliding window, it will lead to erroneous judgments. Considering that the higher the anomaly score, the more obvious the signal fluctuation, we propose a method to evaluate the same period by integrating the scores of multiple streams, which improves the accuracy of detection, as shown in Algorithm 3.
**Algorithm 3:** Anomaly detection.**Input:**    amp_seq**Output:**    time_stamp  1: i← 0;  2: **while** i<***length***(amp_seq)/15 **do**  3:      amp_window ← amp_seq (i× 15+1: i× 15+15);  4:      feature ←$\sigma_{amp\_window}$/$\sigma_{static}$  5:      **if** feature(i) > threshold **then**  6:          j←i+1;  7:        **while** feature(i) > threshold **do**  8:           j++;  9:        **end while**10:      **if** j-1>2 **then**11:         save i, j to time_stamp;12:      **end if**13:      i← j;14:    **else**15:      i++;16:    **end if**17: **end while**

### 3.6. Verification

**Figure 11:**
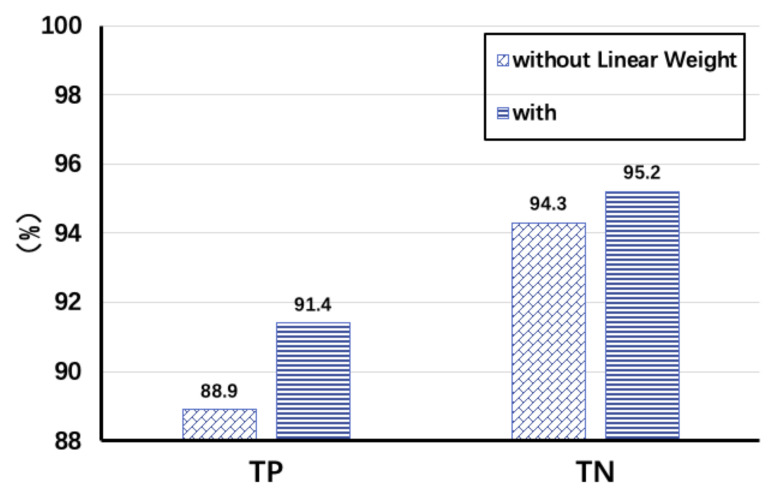
Change of TP/TN for different processing of data.

In this subsection, we will first introduce the methodology of the experiment and then analyze the performance. Two T400 Lenovo laptops with built-in Intel 5300 NICs are used as transceiver devices, and then, the CSI Tool can be used to get real-time CSI data from the driver. The data are collected in the conference room, office, and bedroom corridor, respectively. We use TP (True Positive Rate) and TN (True Negative Rate) to evaluate the performance as shown in Figure 11, where TP represents the correct detection of the presence of moving targets within the sensing range, and TN represents the correct detection of the absence of moving targets within the sensing range.

We first select the size of the sliding window as 0.5 s through extensive experiments. There is another important parameter α, because the value of the static environment features saved in the configuration file is $\hat{F_{k}^{-1}}\left(1-\alpha \right)$, and the value of α affects the environment adaptive mechanism and the anomaly detection. It is found that the missing rate decreases with the decrease of α, while the false detection rate increases slightly, implying that there will be an impact on the sensitivity of the system by α; therefore, we choose α=0.01 by balancing the performance. During the experiment, the data of the target crossing the LoS and nLoS paths are collected in three scenarios, and they show good robustness for different movement speeds. The use of the environment adaptive module and decision refinement module also makes the detection results more accurate, as shown in Figure 11, when Tx and Rx are 5 m apart. TP and TN increase from 88.9% and 94.3% to 91.4% and 95.2% respectively after adding linear weights, and the overall situation of TN is better compared to TP, because there are some missed judgments, especially when the target is far away from the receiver. In addition, we also verify the performance of the dynamic target detection module regarding data diversity. When the target crosses the LoS path, the accuracy of the detection result TP is higher, reaching 92.1%, while when it does not cross, it is only 87.2%, and it reduces to 88.2% when the target moves slowly around 0.3 m/s.

**Figure 12:**
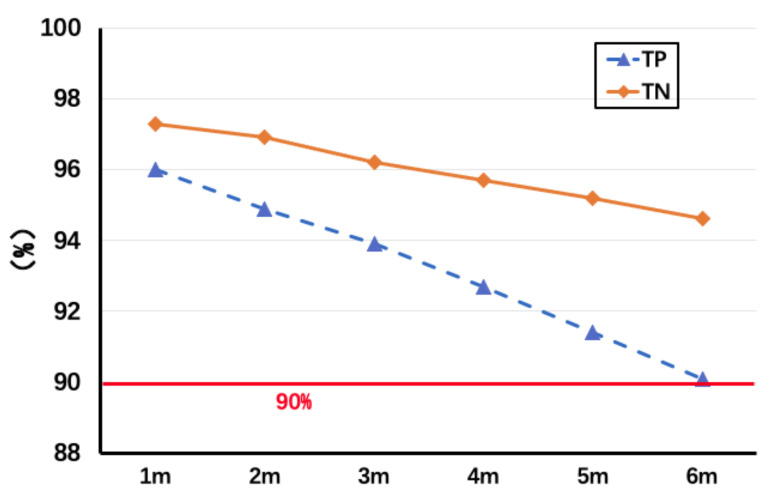
Change of TP/TN with distance between transceivers.

The distance between the Tx and Rx is an important influence on the sensing capability. As Figure 12 shows the change of TP and TN when we adjust the distance between the transceivers from 1 to 6 m, its accuracy will drop rapidly to less than 90% when the distance exceeds 6 m, which we think is an unacceptable ratio. Therefore, we suggest that the distance between Tx and Rx should not exceed 6 m in the actual deployment.

## 4. Rapid Passive Device-Free Tracking

### 4.1. A Wi-Fi Link Grid for Tracking Targets

As depicted above, we can now detect whether a target is present on one link accurately by processing the CSI. Therefore, by the characteristics of the graph, if we have enough links with known locations and specific IDs, depending on the temporal order, we can track how a target has crossed multiple links theoretically.

**Figure 13:**
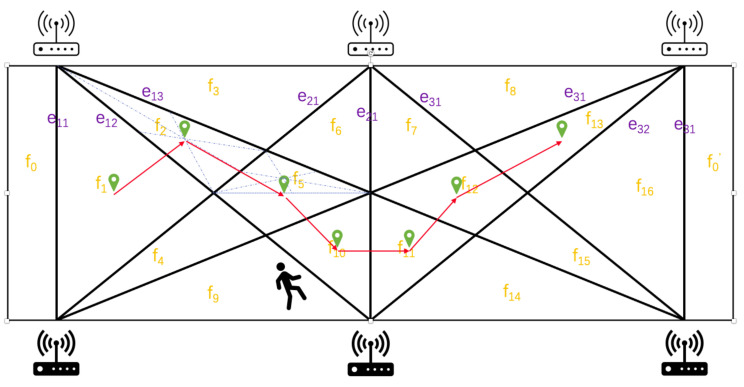
Schematic of the Rapid Passive Device-Free Tracking.

We assume that the graph structure consisting of m,n transceivers is deployed as *G* as in Figure 13. Transceivers are arranged in equal parts on the two long sides of the rectangular room, the room has only one door on the short side, and the position is known. The deployment of transceivers may be more complex in a real environment, but the principle remains the same. We define the vertex set of all the m,n as V(G) and all the edge $e_{ij}$ from m Tx${}_{i}$ to n Rx${}_{j}$ as E(G).

In the WIDE method, we have two basic assumptions:

**Assumption** **1.** *A target’s trajectory is continuous in space and time, which means it does not jump from one place to another.*

In Figure 13, the position passed by a target at two measurement time points must always be adjacent to *G*. For example, from $f_{1}$ to $f_{2}$ in one hop is possible, but from $f_{1}$ to $f_{3}$ is impossible. In other words, any two adjacent locations have at least one common edge.

**Assumption** **2.** *The target can only move through one LoS path of the link at the same time point.*

Based on Assumption 1, we can further assume that a target could only change its location by crossing **one** link. Otherwise, when there is more than one affected link detected at the same time, it may be caused by the joint activity of multiple targets, or it may be caused by a single target that happens to cross the intersection of two links.

The basic principle of our method is the combination of Wi-Fi CSI and an infrared-like grid, which is formed with multiple Wi-Fi links similar to infrared security systems. We take m=n=3 as an example, as shown in Figure 13. We tag all the area as $f_{1},f_{2},\cdots ,f_{16}$, which are partitioned by $e_{ij},0\leq i,j\leq 2$. We assume that the starting position of the target is known, e.g., at the entrance of the room $f_{0}$, and a route of the target is $R:f_{0}\to f_{1}\to f_{2}\to f_{5}\to f_{10}\to f_{11}\to f_{12}\to f_{13}$, in red in the figure, which is used in the rest of the paper as an example. The target will cross $e_{11}$ and enter $f_{1}$ first no matter which direction it will go. Then, if the target goes from $f_{1}$ to $f_{2}$, it must cross the $e_{12}$. Conversely, if the target crosses $e_{11}$ and $e_{12}$ consecutively, the target has only one possible path, $f_{0}\to f_{1}\to f_{2}$. Overall, we use the method described in Section 3 for determining which paths in the grid the target has crossed and then extrapolate backward to get the target’s trajectory based on the order of time.

We use a 9-bit 0–1 code to represent the different positions, and the $0∼8_{th}$ bit from right to left correspond to $e_{11}$ to $e_{13}$, $e_{21}$ to $e_{23}$, and $e_{31}$ to $e_{33}$, respectively. 0–1 indicates the two areas separated by each $e_{ij}$, and we define the part that is nearer to the door $f_{0}$ as 0, and the other part is 1. Therefore, for example, we use 111111111 to represent the location of target when it is in $f_{0}^{\prime }$, and we use 000011111 for $f_{7}$. We can treat the route of a target as a trail on the dual graph $G^{*}$ of *G*. For each move, there will be a one-bit change in theory. When there is a two (or more) bits change, it reveals that the tracking process is wrong and we can correct the error by utilizing the structure of the *G*, which be explained in Section 4.3.

Then, we relax the limitation of the placement of transceivers and consider a more general scenario, and we can get a more complex and common grid. In fact, in reality, the deployment of transceiver devices follows certain rules; for example, AP spacing is roughly the same in the mall ceiling. Even if the selected transceivers are random, the area divided by them is asymmetric; it does not affect the global error. We still use *m* and *n* to represent the number of the transceiver on the two sides. In the best case, each Tx${}_{i}$ and Rx${}_{j}$ forms a different link. Thus, we have the following.

**Property** **1.** *The total number of links of the WIDE method is less than or equal to (m+n)(m+n−1)/2, and the available link is no more than m×n.*

**Proof** **of** **Property** **1.** m+n transceivers form a (m+n)-order complete graph; we assume all the transceivers are activated and duplex, and the number of links is $C_{m+n}^{2}=\frac{1}{2}\left(m+n)(m+n-1\right)$. When they are simplex, they form a bi-graph, and the transmitter and receiver set consists of two sub-graphs, so the number of available links is m×n. □

**Property** **2.** *The number of areas divided by the available links K is no less than m×n.*

**Proof** **of** **Property** **2.** We use mathematical induction to prove the following. First, define $K_{i,j}$ as the number of area when m=i,n=j.

- When m=1,n=1, it is obvious that $K_{1,1}=2>1\times 1$.
- Assume $K_{m_{0},n_{0}}>m_{0}\times n_{0}$ when $m=m_{0},n=n_{0}$.
- When $n=n_{0}+1$, the new $n_{0+1}$ will connect all the $m_{0}$ points and forms $m_{0}$ new links, and $m_{0}\times n_{0}$ intersection points are generated to separate the existed $K_{m_{0},n_{0}}$ areas and get no less than $m_{0}\times n_{0}$ new areas. Therefore, $K_{m_{0},n_{0}+1}\leq K_{m_{0},n_{0}}+m_{0}\times n_{0}>m_{0}\times n_{0}+n_{0}=m_{0}\times \left(n_{0}+1\right)$. Similarly, we get $K_{m_{0}+1,n_{0}}>\left(m_{0}+1\right)\times n_{0}$. Therefore, we get $K_{m_{0}+1,n_{0}+1}>\left(m_{0}+1\right)\times \left(n_{0}+1\right)$.

□

### 4.2. Time Synchronization

As mentioned above, the tracking process needs to have both chronological and spatial information about the target crossing multiple links, so time synchronization (even if it is not strictly synchronized) needs to be guaranteed among different devices. The synchronization we utilize is lightweight.

**Property** **3.** *Any two vertexes of G (Tx${}_{i}$, Rx${}_{j}$, 1≤i,j≤3) could be synchronized in no more than two time slots.*

**Proof** **of** **Property** **3.** *G* is a three-connected graph. The diameter of *G* is d(G)=max{d(u,v)|$u,v\in V\left(G\right)\}=\left\lceil \log_{\Delta -1}\frac{N(\Delta -2)+2}{\Delta }\right\rceil =2$, where the maximum degree Δ=3 and order N=6. Without loss of generality, we assume m>n, so $d\left(G\right)=\left\lceil \log_{m-1}\frac{(m+n)(m-2)+2}{m}\right\rceil <\left\lceil \log_{m-1}\frac{2m(m-2)+2}{m}\right\rceil =\left\lceil \log_{m-1}\frac{2{\left(m-1\right)}^{2}}{m}\right\rceil$. When $m\geq 2,m-1\geq 1,\frac{2}{m}\leq 1,f\left(m\right)=d\left(G\right)$ is monotonically increasing, so $d\left(G\right)<\log_{m-1}{\left(m-1\right)}^{2}=2$. When *m* and *n* take any other value, the same conclusion can be obtained in the same way. □

In addition to this, we set a maximum sync restart time $T_{m}$ for global synchronization.

### 4.3. Self-Correction of the Wrong Tracking Results

As described in Section 3, we can determine the process of a target crossing a Wi-Fi link with about 90% probability by the designed model. Since our method is lightweight, this result is already acceptable. In fact, in practice, even with fingerprinting or training methods, there is no guarantee that the accuracy of the judgment will reach 100%. Can we make up the possible misjudgments by other means?

Inspired by the article [48], we can solve this problem without additional overhead by using the connections in the grid. As depicted above, the target will affect links $e_{12},e_{21},e_{31},\cdots$ in turn in route R. The first condition is that the target is not detected when passing through $e_{21}$, which will lead to a discontinuous route and cannot be tracked. However, when the target continues to move forward, it will affect link $e_{31}$. Based on the condition that the probability of consecutive two misjudges is less than 1%, we can find that there is only one route from $f_{2}$ that passes through only one link and reaches $e_{31}$. So, we can make up the missing paths after just one move.

The other condition is, for example, a target is passing through A, but B is detected, and this condition can be divided into two cases: A and B are both detected or only B is detected, although in practice, the probability of both cases is very small. We still use the above example to explain. First, when the target moves from $f_{2}$ to $f_{5}$, $e_{21}$ is not detected while $e_{13}$ is detected; according to our algorithm, the route will point from $f_{2}$ to $f_{3}$, and when the target continues to move, it will cause $e_{31}$ to be detected; then, it can be found that $f_{3}$ and $e_{31}$ are uncorrelated and separated by at least two links. Based on the above condition that the probability of two consecutive misjudgements is extremely low, we can exclude that the target passes through $e_{31}$ after $f_{3}$ and consider $e_{13}$ as a misjudgment; therefore, we can find the right route to be the same as in the first condition. Second, $e_{21}$ and $e_{13}$ are both detected. If both routes starting from this are consecutive, this may be caused by multiple targets and will be discussed later. Otherwise, the adjacency of the next arrived link to the current location is examined according to the first case of the second condition, thus logically eliminating the wrong route.

The passive tracking of multiple targets is a very big challenge because locking the identity of a target by analyzing the wireless signal usually requires a complex training process, and even just distinguishing whether it is a different person requires a complex signal processing [49,50]. Therefore, most of the current passive tracking efforts are single target. However, our method can solve the multi-target problem to some extent, although it is not perfect. As described in the previous paragraph, if two unrelated links are detected to have fluctuations at the same time or two times very close to each other, $e_{11}$ and $e_{31}$ for example, and both can form a continuous path, that indicates that this is caused by the movement of two targets or even more targets. In case multiple targets do not meet (routes can have intersections), the tracking effect is still the same as for a single target. This is the ideal case where two targets have different starting points. Meanwhile, it is difficult to distinguish when the targets have the same starting point and are moving synchronously, unless at some point, the routes start to separate. Another situation is that two targets with different starting points intersect at a certain point, after which the tracking is still two routes, but the identity of the target can no longer be determined.

### 4.4. Analysis on Tracking Error

The evaluation metrics for all positioning and tracking methods are generally response speed and accuracy. Our proposed method does not require extensive training but relies on data and set thresholds for a simple judgment. As depicted in Section 3.6, the window size is set as 0.5 s to judge whether there is a target crossing the link between transceivers. Therefore, the delay of each move is slightly larger than 0.5 s, which is near real-time progress. As for the error, as shown in Figure 13, we first target some areas that are different in shape and size; however, we need to convert them to a uniform metric that can be generally accepted. We find that most of the $f_{k}$ is a triangle or a simple convex quadrilateral. In geometry, we usually use the center of gravity to represent a polygon. Inspired by this, we take the center of gravity as the coarse-grained location of $f_{k},1\leq k\leq K=16$ here. By connecting these centers of gravity, we can obtain a set of folds to roughly describe the trajectory of the target. However, how to measure the error of it?

We first transfer $f_{k}$ to a round with the same area $s(f_{k})$; then, we take its radius as the localization error of this area denoted as E(k), and we take the average of all localization errors as the global positioning error

(11) $$E\left(k\right)=\sqrt{\frac{s(f_{k})}{\pi }},\;k=1,2,\cdots ,K.$$

The grid is highly symmetrical, satisfying axisymmetric and central symmetry. It is very helpful in improving the uniformity of localization errors, since we have only five different radii when there are 3 × 3 transceivers in Figure 13.

We assume that the side lengths of the rectangular room are *a* and *b*, respectively. Therefore, the areas of $f_{n}$ are $S\left(f_{1},f_{16}\right)=\frac{ab}{8}$, $S\left(f_{2},f_{4},f_{13},f_{15}\right)=\frac{ab}{24}$, $S\left(f_{3},f_{8},f_{9},f_{14}\right)=\frac{ab}{12}$, $S\left(f_{5},f_{12}\right)=\frac{ab}{24}$, $S\left(f_{6},f_{7},f_{10},f_{11}\right)=\frac{ab}{24}$. The radii of the corresponding circles of the same area are $r\left(f_{1,16}\right)=\sqrt{\frac{ab}{8\pi }},\;r\left(f_{2,4,13,15}\right)=\sqrt{\frac{ab}{24\pi }},\;r\left(f_{3,8,9,14}\right)=\sqrt{\frac{ab}{8\pi }},r\left(f_{5,12}\right)=\sqrt{\frac{ab}{24\pi }},\;$ and $\;r\left(f_{6,7,10,11}\right)=\sqrt{\frac{ab}{24\pi }}$. The average tracking error $\bar{E(k)}$ is

(12) $$\bar{E(k)}=\frac{1}{K}\sum_{k=1}^{K}\sqrt{\frac{s(f_{k})}{\pi }}.$$

For example, we assume there is a room with the area of $a\times bm^{2}$; in Figure 13, the average tracking error $\bar{E(k)}$ is

(13) $$\begin{array}{cc} \bar{E(k)} & =\frac{1}{16}\sum_{k=1}^{16}\sqrt{\frac{s(f_{k})}{\pi }}=\frac{1}{16}\left(\sqrt{\frac{ab}{8\pi }}\times 2+\sqrt{\frac{ab}{24\pi }}\times 10+\sqrt{\frac{ab}{12\pi }}\times 4\right)\\ \; & =\frac{1}{16}\sqrt{\frac{ab}{\pi }}\left(\sqrt{\frac{1}{8}}\times 2+\sqrt{\frac{1}{24}}\times 10+\sqrt{\frac{1}{12}}\times 4\right)\\ \; & ≃0.135\times \sqrt{ab}. \end{array}$$

**Figure 14:**
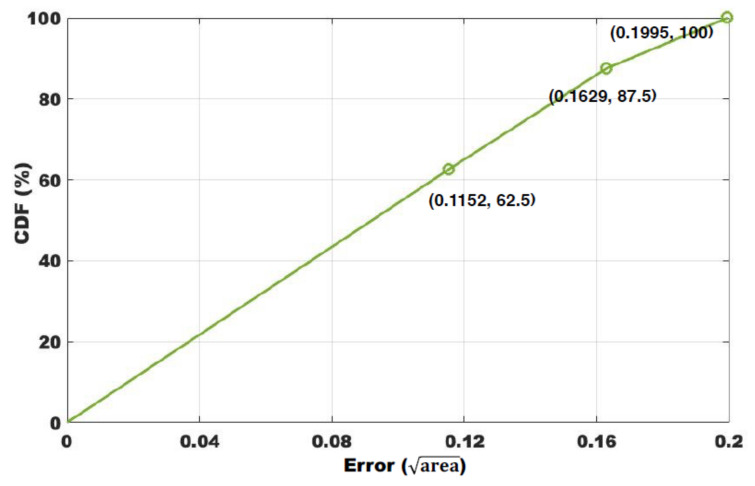
CDF of the error in a a*b room with 3×3 transceivers.

While according to Equations (12) and (13), when the area is 50 m${}^{2}$ (approximate environmental area applied to most works today), the average error is about 0.95 m. The CDF of the error is shown in Figure 14. Another advantage of the WIDE method is that the tracking error is constant once the topology is determined, which is very robust, as long as the detection is reliable for individual links. We show in Table 1 a comparison of some existing tracking methods. It must be admitted that the accuracy of our method is slightly inferior compared to the existing excellent works, but in practice, there should be enough to find the target accordingly. The WIDE method spends a relatively small overhead on signal processing while making full use of the existing network structure, and if the WIDE method is combined with a signal with higher sensing ability such as LoRa, the results will be better and can be applied in more scenarios. More importantly, the features extracted by the WIDE method are easily available on any commercial NIC and accurate, with lower complexity compared to extracting AoA, ToF, and DFS [2,3,51,52], and thus, they are more easily scalable. Therefore, we regard it as a tradeoff between accuracy and overhead.

**Figure 15:**
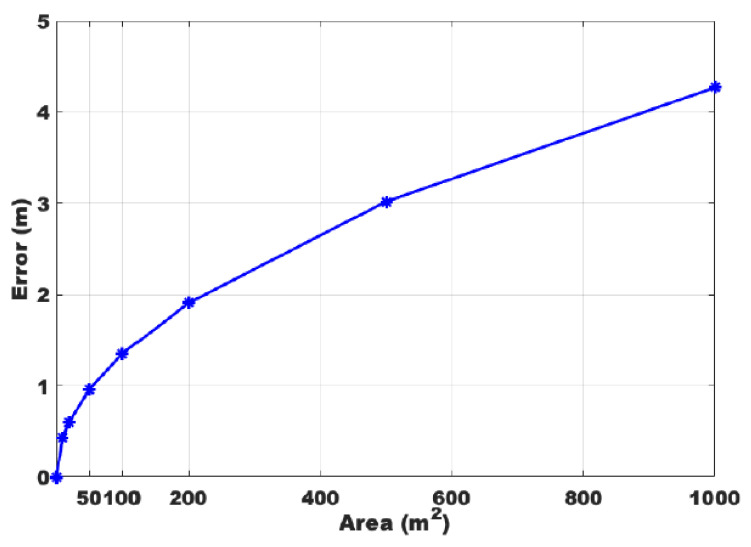
Theoretical value of error variation when using 3×3 as the basic unit.

As the total area increases, the growth rate of the tracking error is slowed down, and the change is very small, as shown in Figure 15.

Due to the limited sensing range of Wi-Fi, the 3×3 deployment used in this paper is an appropriate choice considering the density of the devices; otherwise, two devices at a distance are no longer able to form an effective link. A 3×3 deployment should be used as a basic unit to scale when the monitoring area is large (some devices can be reused; for example, a 4×4 can form two 3×3 s). Since the path is continuous, the endpoint of the route in the 1st area can be regarded as the starting point of the route of the 2nd area, which does not affect the results, which is highly efficient for the reuse of existing equipment. However, the theoretical analysis given above is still applicable in principle to technologies with a larger sensing range, such as using LoRa. In a larger scenario, the error is 1.35 m when the total area is 100 m${}^{2}$, and it is only 4.27 m when the area increases to 1000 m${}^{2}$. If a pair of transceivers is added to 4×4, the error is further reduced to 0.83 m, which is a very desirable tracking result. Finally, we can choose the density of deployment according to the accuracy requirement and the limitation of the number of devices to obtain the desired route error.

## 5. Conclusions and Discussion

In this paper, a Wi-Fi-enabled lightweight passive human tracking method is presented. The WIDE method is invented via analyzing the relationship between moving across the LoS of the transceiver and the physical layer of Wi-Fi signals. The evaluation results showed that the WIDE method allows accurate and near real-time target tracking with a limited number of transceivers. We believe that the WIDE method does not only work well with Wi-Fi devices on the ground. In future work, we plan to solve some more detailed problems, such as the simultaneous occurrence of multiple targets and tracking in 3D environments such as drone detection, etc. Outdoor long-range localization for IoT has been a hard issue because of the complex environment and limited resources. We would like to combine the WIDE method with LoRa and introduce it into larger range tracking. We believe the concept of the WIDE method will also show good performance in the long range.

## Figures and Tables

**Table 1:** A comparison of some existing tracking methods.

Related Work	Accuracy	Experimental Environment	Others
LiFs [3]	0.5 m, 1.1 m	about 100 m${}^{2}$, 11 Tx/Rx	without all Tx/Rx locations
Indotrack [2]	35 cm	6 m × 6 m, 1Tx and 2Rx	high latency
[51]	55 cm	7 m × 7 m, 1Tx and 3Rx	multi-person
[52]	26 cm, 82 cm	7 m × 6 m, 1Tx and 3Rx	single target
WIDE method	0.95 m	50 m${}^{2}$, 6 Tx/Rx	partial multi-target

## Data Availability

Not applicable.
